# Early mother-child relationships in schizophrenic patients hospitalised at the Arrazi psychiatric hospital in Salé

**DOI:** 10.1192/j.eurpsy.2025.897

**Published:** 2025-08-26

**Authors:** N. Ait Bensaid, M. Sabir, F. El Omari

**Affiliations:** 1 arrazi psychiatric hospital, Sale, Morocco; 2 HAS, arrazi psychiatric hospital, Sale, Morocco

## Abstract

**Introduction:**

Schizophrenia is a highly complex mental disorder. It is associated with hallucinations and delusions. Caring for a patient with schizophrenia presents major challenges, especially for mothers. The mother-child relationship is one of the first relationships to be formed, serving as the basis for other human relationships [1,2]. The mother is the most important person shaping the child’s behaviour.

Although schizophrenia has biological and genetic causes, the environment in which the person grew up is very important.

Translated with DeepL.com (free version)

**Objectives:**

To explore the childhood experiences of patients with schizophrenia with their mothers in order to identify the early mother-child relationship in these patients.

**Methods:**

This was a descriptive cross-sectional study using a questionnaire including sociodemographic and clinical criteria as well as questions to assess patients’ childhood experiences with their mothers and patients’ feelings towards their mothers.

Inclusion criteria were patients’ willingness to participate in the study. Participants were male schizophrenic patients over 18 years of age hospitalised at the Arrazi Psychiatric Hospital in Salé.

Exclusion criteria: intellectual disability.

**Key words:**

Schizophrenia, mother-child relationship, emotions, patients.

**Results:**

In this study, a total of 88 male patients with schizophrenia were collected. The majority had an average socio-economic status. Almost 77% were single and 89% lived with their families. All participants were unemployed. 91% had a substance use disorder, including tobacco and cannabis. 77% had reported psychological abuse by their mother and 55% had reported physical abuse. All the participants had received comparisons with other children and the majority reported having already felt that they were going to be abandoned by their mother. 75% reported having played the role of parent for their parents in childhood. 88% of the participants had feelings of anger and blame for their mother, 20% had feelings of security, 51% had ambivalent feelings of love and hate at the same time.

**Conclusions:**

The present study showed that the early relationship between mother and child in patients with schizophrenia was associated with complex and ambivalent emotions and was also characterised by alternating feelings of hatred and love. Feelings of abandonment of the child and lack of attention to the child’s basic needs caused patients many emotional problems towards their mothers.

**Disclosure of Interest:**

None Declared

